# Presence and correlates of autistic traits among patients with social anxiety disorder

**DOI:** 10.3389/fpsyt.2023.1320558

**Published:** 2024-01-19

**Authors:** Barbara Carpita, Benedetta Nardi, Chiara Bonelli, Enrico Massimetti, Giulia Amatori, Ivan Mirko Cremone, Stefano Pini, Liliana Dell’Osso

**Affiliations:** ^1^Department of Clinical and Experimental Medicine, University of Pisa, Pisa, Italy; ^2^North-Western Tuscany Region NHS Local Health Unit, Department of Psychiatry, Pisa, Italy

**Keywords:** social anxiety disorder, social anxiety spectrum, autism spectrum disorder, subthreshold autism spectrum, autistic traits

## Abstract

**Introduction:**

Due to their similar behavioral presentation, it can sometimes be challenging to distinguish between a social anxiety disorder (SAD) and the social avoidance that is frequently described in autism spectrum disorder (ASD). Moreover, a growing body of evidences is reporting that a significant proportion of subjects with ASD also meet the requirements for SAD and, vice versa, subjects with SAD tend to exhibit a higher prevalence of autistic traits.

**Aim:**

In this framework, the current study aims to evaluate prevalence and correlates of autistic traits in a sample of adult subjects diagnosed with SAD and healthy controls (HC), also evaluating which autism spectrum dimensions may statistically predict higher SAD symptoms.

**Methods:**

56 subjects with a clinical diagnosis of SAD and 56 gender and age matched HC were recruited from the Psychiatric Clinic of the University of Pisa. Subjects were assessed with the SCID-5, the Social Anxiety Spectrum – Short Version (SHY- SV) and the Adult Autism Subthreshold Spectrum (AdAS Spectrum).

**Results:**

SAD group scored significantly higher in all AdAS Spectrum and SHY-SV domains and total score compared to the HC group with no significant gender difference. SHY-SV total and domain scores, were strongly and positively and strongly correlated with all AdAS Spectrum domains and total score. AdAS Spectrum total score and *Childhood/Adolescence, Non-Verbal Communication, Empathy* and *Restricted interests and Rumination* domain scores score were significant predictors of higher SHY-SV score.

**Conclusion:**

Our results confirm the link between SAD and autistic traits also in adult population, describing not only high levels of autistic traits in SAD adults, but also significant correlations between many core features of the two disorders and a predictive role of autistic traits on higher SAD symptoms.

## 1 Introduction

Social anxiety disorder (SAD), with a reported lifetime prevalence up to 13% (1, 2) is one of the most prevalent mental illnesses and a topic of significant interest for researchers, although being frequently neglected in clinical settings. According to the Diagnostic and Statistical Manual of Mental Disorders, Fifth Edition – Text Revision (DSM-5-TR), SAD is a syndrome characterized by elevated fear and anxiety that manifest before or during social settings, worries about being poorly perceived, and a propensity to avoid interactions (3). The feelings of fear and anxiety experienced by the patients typically cover a wide range of scenarios: some patients only experience mild symptoms occurring exclusively in specific contexts, while others experience more severe symptoms that interfere with almost all social interactions (e.g., having a conversation or meeting new people, being watched while eating or drinking, performing in front of others) (3, 4). Typically, SAD has an early onset and a chronic course, frequently lasting lifelong (5–7). In most cases, symptoms begin throughout adolescence and last for several years before assistance is sought (8), rising significantly the chances of dropping out of school, failing exams (9), and failing to graduate (10, 11). Moreover, SAD is linked to severe functional disability and noticeably diminished quality of life (9), raising the likelihood of developing another major comorbid disorder such as anxiety and mood disorders and substance or alcohol use disorders throughout the course of the lifespan (12–15).

Furthermore, while SAD can progress and worsen with the development of paranoic delusion, on the other social anxiety has also been reported as a major component in patients with schizophrenia, influencing its treatment, progression and prognosis (16, 17). In particular, the risk of developing depression, is significantly increased by SAD and, in this case, linked to a worse prognosis and a higher chance of suicide attempts (18, 19). In this context, one of the most common co-occurrent disorder reported with SAD is autism spectrum disorder (ASD) (20).

One of the primary concerns in the field of SAD since its conceptualization, has been the establishment of a diagnostic threshold (21). Many researchers indicated that SAD should be categorized as spectrum of severity rather than a discrete condition based on subjectively selected thresholds (22), and that the borders of SAD should be established by severity rather than qualitative traits (23, 24). In this framework, the most recent editions of the DSM introduced some adjustments to the chapter on SAD, reflecting a new and improved knowledge of the disorder in various social contexts (3, 24). Following this idea, some research postulated that SAD should be better classified as a dimensional continuum (22). According to such researches, a spectrum model of psychopathology can help identify the wide range of sub-threshold manifestations that may coexist with the primary mental condition (25). The term “spectrum” is used in this context to characterize mental health illnesses that include a variety of symptoms and behavioral patterns linked to a recognized DSM or ICD construct (25). While the spectrum of symptoms and traits includes the primary symptoms of the current DSM diagnostic categories, it also includes sub-clinical and atypical manifestations, as well as temperamental and/or personality traits and isolated signs and symptoms, symptom clusters, and behavioral patterns (26–32). According to this viewpoint, spectrum symptomatology is similar to the hidden section of an iceberg beneath the water’s surface, whereas full-blown diagnostic criterion symptoms represent only the visible portion (25). Following this model, the “Social Phobia Spectrum Self-report” (SHY-SR) instrument was developed and validated in the early 2000s as part of the “spectrum project,” an international collaboration aimed at shedding light on the validity of a dimensional approach to psychopathology (25–32). The SHY-SR was designed to measure not only the prototypic symptoms of SAD, but also atypical presentations, temperamental features, and other notable clinical and sub-clinical aspects associated with the primary symptoms (33, 34). The questionnaire had a high internal consistency, strong inter-rater reliability, and discriminant validity and throughout the years it has been employed in a variety of clinical contexts throughout the last few decades (7, 33, 35, 36). However, due to the excessively long time for its compilation and some outmoded and superfluous items, recently its application in routine clinical practice has been difficult; for this reason, the same authors recently proposed a shortened and renewed version, the Social Anxiety Spectrum – Short Version (SHY-SV) (37).

ASD is a frequent neurodevelopmental disorder that affect at least 1% of the population (38). The core characteristics of ASD are impaired social communication skills, repetitive and restricted interests and behaviors, and hypo-and hyper-reactivity to sensory stimuli (3). The more recent conceptualization of ASD depicts it as a heterogeneous disorder whose symptoms lie across a continuum that ranges from the milder presentations to most severe ones. In the recent years, research on ASD has emphasized the need to examine milder, sub-clinical manifestations of the autism spectrum as well as the full-blown clinical forms, which appear to be distributed along a continuum from the general to the clinical population (31, 39–42). First-degree relatives of people with ASD were firstly studied for sub-threshold autistic features, which are referred to as “broad autism phenotype” (43, 44); however, additional research has discovered other groups of people that are more likely to exhibit autistic features, from students of scientific courses to psychiatric patients with different types of disorders (44–53). In particular, subthreshold autistic traits are of interest because they exert a negative impact on quality of life and to represent a major risk factor for the emergence of various psychiatric disorders, as well as suicidal thoughts and behaviors (54–57). However, considering that autism spectrum is still a condition mainly associated to child healthcare due to its early onset, subthreshold autistic traits, or even milder clinical forms of ASD may easily remain under-recognized and undertreated during adulthood, silently worsening the trajectory of other comorbid disorders (58).

Due to their similar behavioral presentation, it can sometimes be challenging to distinguish between SAD and the social avoidance that is frequently described in ASD (59, 60). To this date, many researches have demonstrated that the symptoms of SAD and ASD significantly overlap in many fields. The key areas where most of the symptomatologic overlap happens are those of social interaction and social skills (61); for example, the two disorders share the difficulties in talking in front of others (62) and the avoidance of eye contact as well as paying little attention to the area around an interlocutor’s eyes (63). In particular, atypicalities in social attention have been linked to both SAD and ASD. In the first the avoidance of social clues like faces with direct gaze can make it harder for the subject to reappraise or get used to the perceived threat, thus contributing to the maintenance of SAD symptoms (64). In the latter, atypical social attentiveness has been observed to precede clinical signs in ASD (65, 66). A growing body of evidences highlighted that a significant proportion of subjects with ASD also meet the requirements for SAD (67–69), while, in a similar way, subjects with SAD tend to exhibit a higher prevalence of autistic traits (67, 70). In this context, in light due to the relevance of autistic traits even in non-ASD clinical samples, some authors proposed the idea that various psychiatric illnesses might arise as a result of a neurodevelopmental alteration comparable to the one connected to ASD (38, 58, 71, 72), where the vast range of ASD presentations ought to be viewed as the tip of a larger iceberg that contains a number of clinical and non-clinical phenotypes (31, 73).

Accordingly, the recent literature has stressed the necessity to consider disorders like SAD and ASD as extremes along natural continuums phenotypes of traits that are typically distributed throughout the general population, implying the absence of clear-cut borderlines between having no symptoms, increased sub-clinical traits and a formal diagnosis, while even in the latter there are significant individual variances in symptom strength (74–78). Moreover, given the heterogeneity of social competence among the general population, it is plausible that difficulties in social competence functions may link autism spectrum symptoms to increased social anxiety even in non-clinical individuals (79, 80).

In this framework, the aim of the present study was to evaluate prevalence and symptomatologic correlates of autistic traits in a sample of adult subjects diagnosed with SAD and healthy controls (HC), by using two self-report questionnaires, the SHY-SV and the AdAS Spetrum, validated to assess not only the typical symptoms of the two disorders as described by DSM-5-TR criteria, but also the full spectrum of signs and symptoms (so-called atypical as long as they are not included in the diagnostic criteria of the DSM), temperamental traits, and behavioral manifestations of SAD and ASD. Possible autism spectrum dimensions statistically predictive of higher SAD symptoms were also evaluated.

## 2 Methods

Data have been collected between September 2022 and March 2023 at the Psychiatric clinic of the University of Pisa.

### 2.1 Study sample and procedure

The total sample was made of 112 subjects belonging to two diagnostic groups, the SAD group and the HC group, which both counted 56 subjects each. The subjects in the SAD group were recruited from out-patients afferent at the Psychiatric Clinic of the University of Pisa, while subjects in the HC group belonged to health care and paramedical personnel; the recruitment of the latter was carried following a sex-and gender-matched criteria. All subjects had to be between the ages of 18 and 70 and be prepared to sign an informed consent in order to be recruited. The diagnoses of SAD, as well as the lack of mental disorders among HC individuals, were confirmed using the Structured Clinical Interview for DSM-5, Research Version (SCID-5-RV) (81). Exclusion criteria included an age under 18 or over 70, language or intellectual impairment that made the examinations difficult to complete mental disabilities, lack of collaboration skills, and persistent psychotic symptoms.

The presence of BD or Major Depressive Disorder was excluded through the use of the SCID-5-RV and clinical and anamnestic evaluation at the time of recruitment. However, a small number of participants were contemplated to be experiencing a depressive episode as long as the depression symptoms were not as severe as those of the category disorder. Similarly, the existence of other anxiety disorders was acknowledged as long as their symptoms were noticeably less severe than the ones of the SAD.

The study was conducted in accordance with the Declaration of Helsinki. The study was fully explained to the eligible individuals, who then gave their written informed permission after having a chance to ask any questions. The subjects received no compensation for taking part.

### 2.2 Measures

Assessment procedures included the SCID-5-RV (81), the Social Anxiety Spectrum – Short Version questionnaire (SHY-SV) and the Adult Autism Subthreshold Spectrum (AdAS Spectrum). Questionnaire evaluations were carried by psychiatrists, who were trained and certified in the use of the study instruments.

#### 2.2.1 Social anxiety spectrum – short version questionnaire

The SHY-SV consists in 139 items organized in 5 domains (*Interpersonal sensitivity, Behavioral inhibition, Performance, Social situations* and *Substance Abuse*) and an appendix (*Childhood and adolescence*). The answers to the various items are coded in a dichotomous way (yes/no) and the scores relating to the single domains and appendices are obtained by counting the number of positive answers. The SHY-SV was developed by Dell’Osso et al. (37); the validation study reported strong internal consistency, great test–retest reliability and convergent validity with other dimensional measures of SAD.

#### 2.2.2 Adult autism subthreshold spectrum

AdAS Spectrum is a self-report questionnaire designed to assess the wide range of autism spectrum manifestations in adults who do not have language or *intellectual disabilities.* The questionnaire consists of 160 organized into seven domains: *Childhood and adolescence, Verbal communication, Nonverbal communication, Empathy, Inflexibility and Adherence to Routine, Restricted interests and rumination* and *Hyper-and Hyporeactivity to Sensory Input.* The answers to the various items are coded in a dichotomous way (yes/no) and the scores relating to the single domains and appendices are obtained by counting the number of positive answers. The AdAS Spectrum was developed by Dell’Osso et al. (32); the validation study reported a high internal consistency, excellent test–retest reliability (Kunder-Richiardson coeffi-cient = 0.964, ICC = 0.976) and convergent validity with other dimensional measures of autism spectrum (31). The validity and reliability of the questionnaire was confirmed by further studies (39, 40) and a diagnostic threshold was defined at the score of 70.

### 2.3 Statistical analysis

Student’ *t*-test and Chi-square tests were used for comparing socio-demographic variables among group. Student’ *t*-test was used to compare the scores obtained by the two diagnostic groups on the SHY-SV and AdAS questionnaires as well as to compare the scores obtained in the two questionnaires in the SAD group divided by gender. Pearson’s correlation coefficient was used for evaluating the pattern of correlations among the scores reported on the two psychometric instruments within the SAD, and HC subjects. Subsequently, in order to evaluate which AdAS Spectrum domains were statistically predictive of SHY-SV score in the sample, linear regression analyses were performed with SHY-SV scores as the dependent variable and AdAS Spectrum total (first regression) and domain scores (second regression) as independent variables. All statistical analyses were performed with SPSS version 26.0 (82).

## 3 Results

The SAD sample reported a mean age of 39.57 years (±12.56) and consisted of 29 (51.8%) males and 27 (48.2%) females. The HC group reported a mean age of 38.52 years (±13.05) and consisted of 25 (44.6%) males and 31 (55.4%) females (see Table 1). Regarding the educational level, 14 (12.5%) subjects reported to have a master degree, 39 (38.4%) subjects reported to have a degree, 47 (42%) subjects referred to have graduated, 10 (8.9%) subjects had a middle school certificate, 1 (0.9) subject did not finish the elementary school and 1 subject preferred not to disclose. Regarding the occupational role, 23 (20.5%) subjects were students, 19 (17%) subjects were unoccupied, 19 (17%) subjects were housewives, 35 (31.3%) subjects were employed, 1 (0.9%) subject was retired and 15 (13.4%) subjects preferred not to disclose. Regarding the marital status 13 (11.6) subjects referred to live with their parents, 19 (17%) subjects were married, 29 (25.9%) subjects were unmarried, 6 (5.4%) subjects were divorced, 1 (0.9%) subject was a widower and 44 (39.2%) subjects preferred not to disclose.

**Table 1:** Age and sex in the overall sample and comparison between diagnostic groups.

	SAD	HC		
	mean ± SD	mean ± SD	*t*	*p*
Age	39.57 ± 12.56	38.52 ± 13.05	0.435	0.664

		*n* (%)	*n* (%)	Chi-square	*p*
Sex	M	29 (51.8) ^a^	25 (44.6) ^a^	0.449	0.285
	F	27 (48.2) ^a^	31 (55.4) ^a^		

Each subscript letter denotes a subset of categories whose column proportions do not differ significantly from each other at the 0.05 level.

Student *t*-test results showed that SAD group scored significantly higher in all AdAS Spectrum and SHY-SV domains as well as in their total compared to the HC group (see Tables 2, 3).

**Table 2:** Comparison of SHY-SV scores among the diagnostic groups.

SHY-SV scores	SAD group mean ± SD	HC group mean ± SD	*t*	*p*
Interpersonal sensitivity	14.59 ± 4.60	1.66 ± 2.87	17.83	<0.001
Behavioral inhibition	6.70 ± 2.82	0.73 ± 1.29	14.39	<0.001
Social situations	21.39 ± 5.50	2.03 ± 2.06	24.65	<0.001
Aubstance abuse	1.41 ± 1.42	0.21 ± 0.45	5.99	<0.001
Performance	14.78 ± 5.58	1.32 ± 1.86	17.13	<0.001
Total	58.87 ± 12.35	5.96 ± 6.99	27.90	<0.001

**Table 3:** Comparison of SHY-SV scores among the diagnostic groups.

AdAS spectrum scores	SAD group mean ± SD	HC group mean ± SD	*t*	*p*
Childhood/Adolescence	10.43 ± 8.24	0.66 ± 1.12	8.79	<0.001
Verbal communication	7.70 ± 4.19	0.46 ± 0.60	12.80	<0.001
Non-verbal communication	11.68 ± 5.90	1.00 ± 1.17	13.28	<0.001
Empathy	4.96 ± 3.01	0.20 ± 0.48	11.71	<0.001
Inflexibility and adherence to routine	11.75 ± 6.10	1.45 ± 1.65	12.20	<0.001
Restricted interest and rumination	5.91 ± 3.77	0.86 ± 1.34	9.46	<0.001
Hyper– and hyporeactivity to sensory input	3.80 ± 4.32	0.30 ± 0.57	6.01	<0.001
Total Score	56.23 ± 19.89	4.93 ± 4.25	18.87	<0.001

Moreover, results from the comparison between gender in the SAD group highlighted no significative difference in the scores obtained on the AdAS Spectrum (see Table 4).

**Table 4:** Comparison of AdAS spectrum scores between males and females in the SAD group.

AdAS scores	*M* mean ± SD	*F* mean ± SD	*t*	*p*
Child./Adolesc.	9.14 ± 3.85	11.81 ± 11.13	−1.186	0.245
Verb. comm.	8.45 ± 3.54	6.89 ± 4.72	1.405	0.166
Non Verb. comm.	12.21 ± 4.12	11.11 ± 7.40	0.678	0.502
Empathy	4.31 ± 2.04	5.67 ± 3.70	−1.682	0.100
Inflex. & routine	10.96 ± 4.99	12.59 ± 7.11	−0.997	0.323
Restrict. interest & rum.	5.07 ± 3.00	6.81 ± 4.32	−1.766	0.083
Hyper-hyporeact.	3.59 ± 4.43	4.04 ± 4.27	−0.387	0.700
Total	53.72 ± 13.63	58.92 ± 24.94	−0.959	0.344

According to the correlation analysis, in the total sample, the total SHY-SV scores, as well as all SHY-SV domains scores, were significantly and positively correlated with all AdAS Spectrum domain and AdAS total score. All correlation coefficients were strong, with the exception of those reported for the SHY-SV domain Substance abuse, which were medium or weak (see Table 5).

**Table 5:** Pearson’s correlations coefficients (*r*) among SHY-SV domains score and AdAS spectrum scores in the total sample.

	Child./Adolesc.	Verb. comm.	Non-verb. Comm.	Empathy	Inflex. & routine	Restrict. interest & rum.	Hyper-hyporeact.	Tot. score
Interpers. sens.	0.592*	0.762*	0.702*	0.657*	0.697*	0.641*	0.581*	0.823*
Behav. inhib.	0.609*	0.690*	0.702*	0.612*	0.623*	0.508*	0.621*	0.781*
Social sit.	0.623*	0.760*	0.815*	0.783*	0.734*	0.684*	0.513*	0.876*
Subst. abuse	0.191*	0.625*	0.566*	0.435*	0.343*	0.347*	0.217*	0.478*
Performance	0.526*	0.711*	0.763*	0.634*	0.655*	0.716*	0.341*	0.780*
Tot. score	0.624*	0.801*	0.819*	0.741*	0.737*	0.706*	0.531*	0.885*

* Significant correlation for *p* < 0.05.

Lastly, results from linear regression analysis showed that AdAS Spectrum total score was a significant predictor of a higher SHY-SV score (beta = 0.88; *t* = 19.92; *p* < 0.001) (see Table 6). A further linear regression highlighted the AdAS Spectrum *Childhood/Adolescence* (beta = 0.12; *t* = 2.19; *p* = 0.031)*, Non Verbal Communication* (beta = 0.35; *t* = 4.28; *p* < 0.001)*, Empathy* (beta = 0.26; *t* = 4.54; *p* < 0.001)*, Restricted interests and Rumination* (beta = 0.21; *t* = 3.10; *p* = 0.002) domain scores as significant positive predictors of high SHY-SV total scores (see Table 7).

**Table 6:** Linear regression analysis with SHY-SV total score as a dependent variable and AdAS Spectrum total score as independent variables in the total sample.

	b (SE)	BETA	*t*	*p*	CI 95%	
					Lower bound	Upper bound
*constant*	6.36 (1.81)		3.507	0.001	2.766	9.954	AdAS spectrumtotal score	0.85 (0.04)	0.885	19.917	<0.001*	0.767	0.937

R square = 0.783; Adjusted R square = 0.781. *= statistically significant value (*p* < 0.05).

**Table 7:** Linear regression analysis with SHY-SV total score as a dependent variable and AdAS spectrum domains as independent variables in the total sample.

	b (SE)	BETA	*t*	*p*	CI 95%	
					Lower bound	Upper bound
*constant*	5.77 (1.66)	–	3.479	0.001	2.483	9.063
Child./Adolesc.	0.56 (0.21)	0.124	2.193	0.031*	0.044	0.876
Verb. comm.	0.90 (0.49)	0.149	1.853	0.067	−0.064	1.871
Non Verb. comm.	1.46 (0.34)	0.351	4.279	<0.001*	0.782	2.132
Empathy	2.28 (0.50)	0.259	4.543	<0.001*	1.287	3.281
Inflex. & routine	−0.27 (0.35)	−0.065	−0.770	0.443	−0.960	0.423
Restrict. interest & rum.	1.60 (0.51)	0.214	3.104	0.002*	0.578	2.622
Hyper-hyporeact.	0.798 (0.41)	0.099	1.937	0.055	−0.019	1.615

R square = 0.832; Adjusted R square = 0.820. *= statistically significant value (*p* < 0.05).

## 4 Discussion

According to our results, the group of subjects diagnosed with SAD not only scored, as expected, significantly higher in the SHY-SV questionnaire than the HC group, but also reported a higher prevalence of positive answers in all AdAS Spectrum domains as well as in the questionnaire’s total score with no significant difference when compared by gender. Moreover, we globally found significant and strong positive correlations between SHY-SV and AdAS Spectrum domain scores.

Our results are in line with previous studies, confirming the significant presence of autistic traits in adults with SAD. Previous researches, mainly conducted in children, adolescents or young adults, while exploring the relationship between ASD and SAD in clinical and non-clinical samples (83–85) reported that the symptoms of SAD and ASD significantly overlap in many fields, to the point that it can sometimes be challenging to distinguish between SAD and the social avoidance that is frequently described in ASD (58, 60). The recent literature reported how the symptomatologic overlap between the two conditions has been mainly identified, in children and adolescents, in the areas of social interaction and social skills (61), including difficulties in talking in front of others (62) and the avoidance of eye contact (63). Moreover, many studies highlighted not only that traits of both disorders can co-occur and are highly correlated, but also that the correlation is not exclusively due to the symptomatologic overlap between the disorders, nor to artefacts of measurement errors. Additionally, alongside the evidences of a high prevalence of SAD in ASD subjects (67–70) and similarly of autistic traits in SAD patients (67, 70), some studies reported how autistic traits and SAD symptoms are correlated even in subjects that do not meet the full diagnostic criteria while, in neurotypical children, higher autistic traits have been associated with a greater risk of developing SAD later in life (86).

According to our results, the AdAS Spectrum total score and AdAS Spectrum *Childhood/Adolescence, Non Verbal Communication,* the *Empathy* and the *Restricted interests and Rumination* domains were significant positive predictors of higher scores in the SHY-SV. While previous studies in children and adolescents also reported how greater autistic traits significantly predict higher SAD symptoms (60, 68), the finding of *Non Verbal communication* and *Empathy* domain scores being positive predictors of SAD is consistent with a large body of research that identified in deficits of social competence the main common background for the relationship between SAD and autistic traits (80). Indeed, subjects with elevated autistic traits are more likely to have lower social skills, which can be shown as difficulties in social interactions with their peers as well as a higher vulnerability to bullying and victimization (87). Similarly, reduced social competence may predict, also in neurotypical subjects, negative changes in peer interactions and stressful social experiences, playing a crucial role in the development of SAD (88–90). According to other authors, the combination of increased physiological arousal and decreased social competence, significantly predicted social anxiety symptoms in children with and without ASD (89, 90). Furthermore, somewhat in line with our finding of a predictive role of impaired empathy skills for SAD symptoms, studies that focused on evaluating the Theory of Mind (ToM) in SAD samples reported how higher levels of SAD were related to reduced ToM ability (91–96) and empathic accuracy, especially for positive stimuli (91). As a matter of fact, the use of strategies of social camouflaging have been reported among autistic people in order to cope with social difficulties: while the use of these strategies may actually improve social functioning, it was associated with higher anxiety and depressive symptoms (47). Subjects with high functioning ASD or autistic traits may be more aware of their social incompetence than subjects with more severe forms of ASD, thus more frequently developing SAD symptoms. At the same time, the increased tendency towards ruminative thinking may further increase the risk of brooding about social difficulties and fear of judgment (58, 97). In this context, in light of recent findings that highlighted how the presence of higher autistic traits predicts worse treatment results, it is crucial to understand the mechanisms and processes through which ASD symptoms increase the risk of co-occurring social anxiety symptoms (98, 99). Social competence is a skill that both typically developing children (100) and children with ASD (101) can learn with proper support; therefore, it is critical to investigate how emphasizing social competence development in all children, and particularly in those with neuroatypical traits, may reduce the risk for future distress and impairment, such as increased social anxiety. Social competence has been previously identified as a suitable intervention target to reduce overall social anxiety symptoms both in the clinical and non-clinical population (102, 103). Additionally, it could be a crucial target for the prevention of SAD also among subjects with elevated autistic traits, in line with most first-line psychosocial treatments for ASD (103), but also in neurotypical subjects and even those with psychiatric disorders other than ASD (74). Due to the continuous distribution of autistic and social anxiety traits in the general population, disorders like SAD and ASD could be better conceptualized as extremes along continuum phenotypes of traits, implying the absence of clear-cut borderlines between having no symptoms, increased sub-clinical traits and a formal diagnosis (74–78, 104, 105).

This study should be considered in light of important limitations. First of all, the cross-sectional design prevented us to make inferences about temporal or causal relationship between the investigated variables. In addition, Moreover, the relatively small sample size limits the extensibility of our results. Globally, more studies, possibly with a longitudinal design are needed to clarify the role of underlying autistic traits in the development and militainment of SAD symptoms.

## 5 Conclusion

In conclusion, our results confirm the link between SAD and autistic traits also in adult population, describing not only high levels of autistic traits in SAD adults, but also significant correlation between many core features of the two disorders and a predictive role of autistic traits on higher SAD symptoms. This data may support the hypothesis of a possible neurodevelopmental basis for different psychiatric conditions: in particular, several psychiatric illnesses may arise as a result of a neurodevelopmental alteration similar to the one associated with ASD (33, 52, 65) where the wide range of ASD manifestations can be seen as the tip of a wider iceberg featuring clinical and non-clinical phenotypes. In this framework, as hypothesized for eating disorders, SAD could also be conceptualized as a specific phenotype of the autism spectrum (93). On this basis and in light of the now recognized existence of an ASD presentation unique to females that differ from the typical male conceptualization, future researches should also delve deeper into the possibility of considering some disorders as gender influenced autistic phenotypes.

## Data availability statement

The raw data supporting the conclusions of this article will be made available by the authors, without undue reservation.

## Ethics statement

The studies involving humans were approved by Ethical Committee of Azienda Ospedaliera Universitaria Pisana. The studies were conducted in accordance with the local legislation and institutional requirements. The participants provided their written informed consent to participate in this study.

## Author contributions

BC: Conceptualization, Supervision, Writing – original draft, Writing – review & editing. BN: Investigation, Writing – original draft, Writing – review & editing. CB: Investigation, Writing – review & editing. EM: Formal analysis, Writing – review & editing. GA: Investigation, Writing – review & editing. IC: Conceptualization, Supervision, Writing – review & editing. SP: Conceptualization, Supervision, Writing – review & editing. LD: Conceptualization, Supervision, Writing – review & editing.
